# Report of the Committee of the American Gynecological Society

**Published:** 1910-06

**Authors:** 


### TEACHING OF OBSTETRICS

### Report of the Committee of the American Gynecological Society

### Present Status of Obstetrical Education in Europe and America, and Recommendations for the Improvement of Obstetrical Teaching in America

*President of the American Gynecological Society and Fellows:*

Your committee has received reports from Great Britain, Germany, Austria, Switzerland, France and Italy. In contrast with the present system in those countries, a report is submitted from seven representative medical schools in the United States, which may be fairly classed among the best medical schools in this country.

GREAT BRITAIN.

A course of lectures, thirty to forty or more each year, is given in obstetrics in all London schools. It usually extends over two years and lectures on gynecology are given at many schools in addition to those in obstetrics. You will find details as to hours in the *British Medical Journal* for September 4, 1909.

The work in obstetrics consists of the above lectures, clinical teaching in the obstetrical wards (most of the general hospitals now have beds for this, numbering from eight to twelve). A class of practical obstetrics, demonstrations in the museum, personal attendance on about fifty cases each student, the number varying with the different hospitals. Each student must attend twenty cases and in addition each university student (Oxford and Cambridge), must have previously attended cases in the lying-in wards for at least one month.

The teachers of obstetrics also teach diseases of women and their surgical treatment; they are the only teachers who do teach this subject in the medical schools for men students.

(Signed) HERBERT SPENCER.

GERMANY.

I have arranged the instruction in obstetrics and gynecology in the University of Königsberg as follows:

*Sixth Semester.*—Theoretic obstetrics.

*Seventh Semester.*—Obstetrical-gynecological clinic (as spectator); a course in gynecological diagnosis. A course in examinations of pregnant women.

*Eighth Semester.*—Obstetrical-gynecological clinic (as practitioner). A course on obstetrical operations on the mannikin.

*Ninth Semester.*—Obstetrical-gynecological clinic (as practitioner). A course in microscopical diagnosis. A practical course in minor gynecological therapeutics. The physiology and pathology of the new-born infant.

*Tenth Semester.*—Obstetrical-gynecological clinic. Course in obstetrical operations. Course in cystoscopy. Physiology and pathology of the puerperium. A demonstration, weekly, for nine weeks of pathological anatomy (with the epidiascope, microscope, and the like).

Each student in the tenth semester must live a month in the clinic where he observes and conducts about forty labors and performs the minor operations.

(Signed) PROFESSOR WINTER.

AUSTRIA.

Of the five years course, the student must occupy himself during one year with obstetrics and gynecology. During this time he is obliged to attend the lectures ten hours a week. During this time also he must have his practical training in which he has the opportunity to see a large number of labors and to perform minor operations such as perineal lacerations, episeotomy, manual extractions, and the like.

There is mannikin practice in the obstetrical operations.

In addition, he receives practical training in the examination of pregnant women, and gynecological patients. The examination consists of diagnosis in parturient and pregnant women and in gynecological patients and operations performed upon the mannikin.

HEINRICH PEHAM,

University Professor of Obstetrics and Gynecology, Vienna.

### SWITZERLAND.

1. During the customary ten semester medical course, three to four semesters are devoted to obstetrics and gynecology. Three semesters are obligatory.

2. During this time, the students visit the obstetrical-gynecological clinic and polyclinic where opportunity is afforded them to observe gynecological cases, to examine pregnant women and thus to acquire the necessary technical skill.

In addition, a certain proportion of the students attend the theoretical lectures on obstetrics and gynecology, which are not obligatory.

The obstetrical operations are practised upon the mannikin and in addition the students occasionally have the opportunity to perform these operations upon the living patient under the supervision of an instructor.

In the final examination, there is required:

1. Practical demonstration of sufficient knowledge in the examination of pregnant and parturient women and of gynecological patients.

2. The performance of several obstetrical operations on the mannikin.

3. A theoretical oral examination on obstetrics and gynecology.

TH. WYDER,

Director of the University Frauenklinik, Zürich.

### FRANCE.

In answer to your letter of November 26, I went to see Professor Lannelonge, one of the leading surgeons here, also a member of the "Institute" of France and senator. The following is a translation of the answers he dictated to me after reading the questions of your letter:

"Two terms of six months each are devoted to the study of midwifery and obstetrics. The students of the two clinical departments are inscribed turn about night and day to make a stage in the hospital wards and follow the labor hour by hour till period of delivery. During a term they can follow about fifteen cases or more if they wish to do so.

"The scope of the course in obstetrics includes not only delivery proper but also all the medical or surgical treatment of women's diseases such as, for example, fibromes, disease of the ovaries, of the large ligaments, and the like.

"In France the courses are no more given in a theoretical way but are principally practical demonstrations either in the lecture rooms or in the hospitals (woman's wards). All apparatus or instruments for demonstration are used, mannikin work, ward work, polyclinic service, touch courses, and the like.

"In one word the teaching is very complete and great stress is laid on the assiduity of candidates. One can say that after their two terms of practically a year's duration, the students are quite qualified to undertake any kind of delivery and have a sufficient knowledge of women's diseases from a practical view as from a scientific one. This study being far from neglected."

### ITALY.

In Italy there are schools for obstetrics and gynecology for physicians annexed to all the universities. Equally in all the universities are annexed schools for mid-wives. In Florence, there is the Superior Institute for obstetricians and physicians.

The course of obstetrics is of one year for the physicians (the full university course for physicians is six years) and the course of obstetrics is by rule assigned at the sixth year For midwives the course is of two years.

The character of teaching is theoretic and experimental (clinic) and comprises also the assistance of women in labor made by the teachers or by their assistants.

The course includes also diseases of women and their operative treatment, as well as the physiology and pathology of the child-bearing process.

The theoretical instruction is given three times a week for the students in medicine, while it is daily for the midwives. The clinic practice is daily for everybody.

The students in medicine and the midwives cannot perform any operation before the end of their course of studies.

The examination is only theoretic.

[*Concluded in June Number*]

---

Little Willie—Say, pa, what is foresight?

Pa—Foresight, my son, is the faculty of being around when there is a melon to be cut.—*Chicago News.*

